# Determination of Colistin Resistance in Clinical Isolates from Healthcare Facilities in Mthatha and Surrounding Areas

**DOI:** 10.3390/antibiotics14050505

**Published:** 2025-05-14

**Authors:** Silindokuhle Ndlela, Ravesh Singh, Sandeep Vasaikar

**Affiliations:** 1 Division of Medical Microbiology, Department of Laboratory and Pathology, Walter Sisulu University, Mthatha 5117, Eastern Cape, South Africa; silindokuhlen1@gmail.com; 2 Department of Medical Microbiology, University of KwaZulu Natal, Durban 4013, KwaZulu-Natal, South Africa; singhra@ukzn.ac.za

**Keywords:** carbapenem-resistant *Enterobacterales* (CRE), carbapenems, colistin, mobile colistin resistance (*mcr*), CRE infections, carbapenemase genes

## Abstract

**Background**: Antimicrobial resistance (AMR) is a global threat in the public healthcare sector. The emergence of carbapenem-resistant *Enterobacterales* (CRE) has become a serious public health threat in South Africa. The spread of CRE has led to the use of colistin for treating severe infections. Colistin is a cationic, lipopeptide antibacterial agent that is effective against most Gram-negative bacteria through its disruption of the bacterial cell membrane. This study aims to determine the colistin resistance (MIC) and mobile colistin resistance (*mcr-1*) gene in clinical isolates from healthcare facilities in Mthatha and its surrounding areas. **Methods**: Fifty-three CRE isolates were collected from health facilities between January 2019 and June 2021 and stored in skim milk 10% and 5% inositol broth. The carbapenemase confirmatory test involved a RESIST-4 O.K.N.V assay (Coris BioConcept, Gembloux, Belgium), which was conducted following manufacturer protocol. Broth microdilution was performed according to the ISO standard method (20776-1) using A ComAspTM colistin 0.25–16 μg/mL MIC Broth. Conventional polymerase reaction (PCR) was performed for the detection of *mcr-1*. **Results**: *N* = 53 (100%) isolates were used. A total of 53% were defined as *Klebsiella pneumoniae*, Escherichia coli constituted 8%, *Enterobacter cloacae* 8%, *Serratia marcescens* 8%, *Serratia fonticola* 2%, *Enterobacter aerogenes* 2%, *Klebsiella oxytoca* 2%, *Citrobacter koseri* 2%, and *Citrobacter freundii* 2%. The specimens were from the following wards: Pediatric and Neonatal 38%, Medical 30%, Gynecology, Labour, and Maternity 11%, OPD and A&E 11%, ENT 4%, and Others—Male TB ward, Trauma, and adult ICU 6%. In total, 13% of the isolates were resistant and 86% were sensitive to colistin. The common CRE genes detected were OXA-48 at 47%, NDM at 13%, VIM at 1%, and a combination of OXA-48 and NDM at 5%. Of the isolates, 66% were positive for the production of carbapenamase. In this study, we found that all *N* = 53 (100%) isolates did not have the mobile colistin resistance gene (*mcr-1*). **Conclusions**: Antimicrobial resistance is associated with the emergence of carbapenemases genes. Increasing resistance to colistin in clinical settings can lead to difficulties in treating CRE infections, which may lead to clinical failure. In our study, 13% of isolates were phenotypically resistant to colistin.

## 1. Introduction

The Centers for Disease Control and Prevention consider carbapenem-resistant *Enterobacterales* (CRE) as an urgent public health threat [1]. The global spread of multidrug-resistant (MDR) CRE has led to the use of colistin for treating severe infections [2]. Colistin is considered a last resort antibiotic for treating CRE infections [3]. Colistin is a cationic, multicomponent lipopeptide antibacterial agent that is effective against most Gram-negative bacteria (including *Acinetobacter*, *Pseudomonas*, *Escherichia*, and *Klebsiella* species) by disrupting the bacterial cell membrane [4].

### Resurgence of Colistin

In the past, colistin was disregarded due to concerns regarding nephrotoxicity and neurotoxicity [5]. Colistin was re-introduced in clinical practice in the 2000s as it retains activity against multidrug-resistant (MDR) and extensively drug-resistant (XDR) Gram-negative pathogens, including carbapenemase-producing isolates [6]. Colistin is a concentration-dependent bactericidal antibiotic. The resurgence of colistin as an antimicrobial therapy is a result of the lack of available treatments for MDR bacteria, including *Acinetobacter baumannii*, *Pseudomonas aeruginosa*, and *Klebsiella pneumoniae* [7]. It is one of the few remaining treatments for infections caused by carbapenem-resistant *Enterobacterales* (CRE) and other MDR pathogens. Significant misunderstanding has persisted since colistin was brought back into the clinic because of the various conventions used to describe the dosages of the polymyxins, variations in their formulations, out-of-date product information, and uncertainties regarding susceptibility testing. As a result, it is unclear how best to use and dose colistin and polymyxin B. A guide for colistin therapy and its optimal clinical use is provided by international consensus recommendations [8,9].

Colistin disrupts the bacterial outer membrane by binding to lipopolysaccharides (LPS) and increases cell permeability, leading to cell death [10]. The use of colistin is challenged by the emergence of chromosomal and plasmid-borne colistin resistance [11]. Transposable genetic elements (plasmids with the *mcr* genes) are the major cause of bacterial colistin resistance in the microbial world. Ten variants of the mobilized colistin resistance genes, *mcr 1–10*, have been identified [12].

There is a need for careful dosing strategies to balance therapeutic efficacy with minimizing potential toxicity. It is for this reason that a professional healthcare worker must monitor the use of colistin, and pediatric and adult dosing guidelines must be followed. Colistin is a Schedule 21 drug [9]. Only licensed pharmacies can dispense Schedule 21 drugs and must adhere to regulations to ensure that these drugs are used appropriately. The reliance on colistin is a big challenge for clinicians, since the overuse of this drug will eventually lead to resistance. There are reports on the emergence of colistin resistance worldwide [13].

The emergence of the *mcr-1* gene reported in one of the most recent publications restricts treatment options, particularly in the case of *Klebsiella pneumoniae* (CRKP) and other CRE strains. *mcr-1* has now been detected in clinical isolates of colistin-resistant *E. coli* from hospitalized (*n* = 3) and outpatient-based (*n* = 6) patients in SA [14]. Therefore, there is a need for strict antimicrobial stewardship to preserve the efficacy of colistin and other last-line antibiotics.

Other mechanisms of colistin resistance are the two-component systems (TCS) of *PmrAB* and *PhoPQ* and the *mgrB* gene, and the point mutations in the *pmrA/B*, *phoP/Q*, *mgrB*, and *crrB* genes and the *mcr-4.3* gene. Also, the efflux pump and capsule formation add to the resistance [15,16].

The continued spread of carbapenemases would result in a clinical catastrophe and could cause a future public health crisis. This study aims to determine the most common gene responsible for colistin resistance and the susceptibility of CRE to colistin. Studies have since reported the occurrence of *mcr* genes in various parts of the world, raising alarm about colistin’s diminishing efficacy as a last-resort antibiotic.

## 2. Results

### Participant’s Eligibility Inclusion and Exclusion

A total of 53 viable CRE isolates were analyzed during the experimental investigation. The distribution of different CRE isolates was identified using the BioMérieux Vitek-2 system. *Klebsiella pneumoniae* was the most frequently isolated species *N* = 31 (53%), followed by *Enterobacter cloacae N* = 9 (17%), *Escherichia coli* and *Serratia marcescens N* = 4 (8%), and *Serratia fonticola*, *Enterobacter aerogenes*, *Klebsiella oxytoca*, *Citrobacter koseri*, and *Citrobacter freundii N* = 1 (2%) (Table 1).

**Figure 1:**
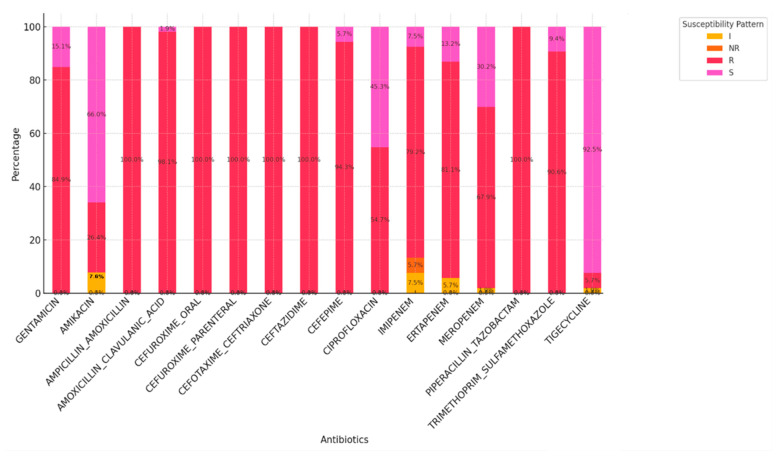
Antimicrobial profile of CRE isolates based on BioMérieux Vitek-2 system.

The antimicrobial profiles of the isolates in Figure 1 were also analyzed using BioMérieux Vitek-2 in the NHLs, Mthatha. Carbapenem resistance was recorded at 94.3% with cefepime, 79.2% with imipenem, 81.1% with ertapenem, and 67.9% with meropenem.

**Figure 2:**
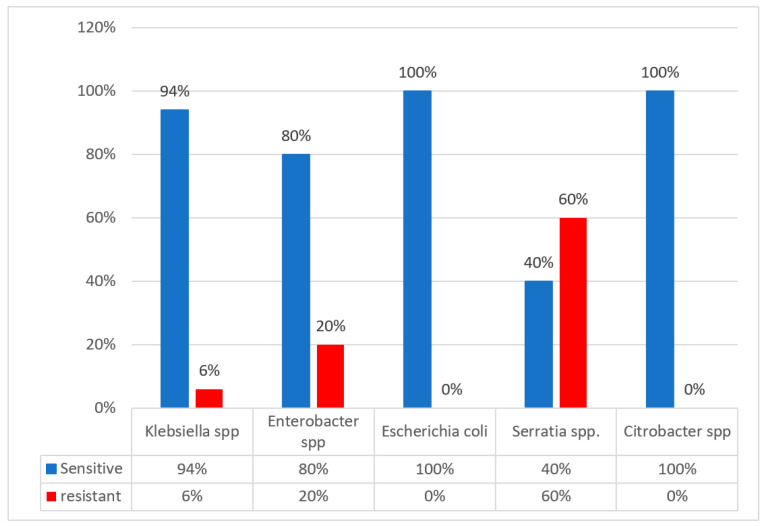
The sensitivity of CRE isolates on colistin using the broth microdilution Method.

The broth microdilution method determines the minimum inhibitory concentration (MIC), the lowest colistin concentration that inhibits microorganisms’ growth. This method was used to determine colistin MIC; Figure 2 shows the proportion of colistin resistance obtained. A total of 6% of *Klebsiella* species were resistant to colistin, followed by 20% *Enterobacter* species and 60% of *Serratia* species.

**Figure 3:**
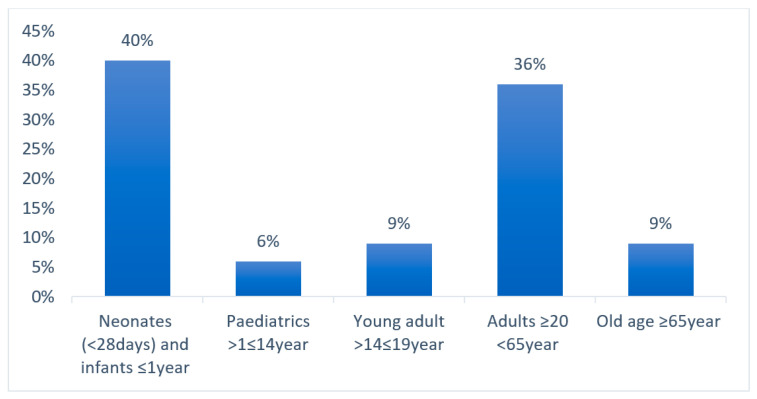
Distribution of CRE cases in different age groups.

**Figure 4:**
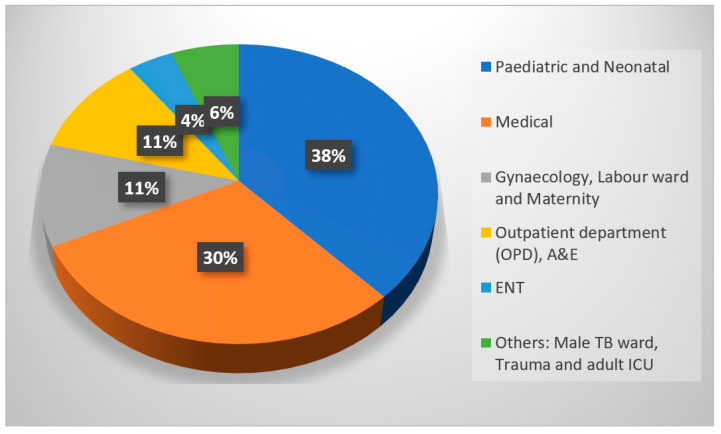
Distribution of CRE cases in different wards.

**Figure 5:**
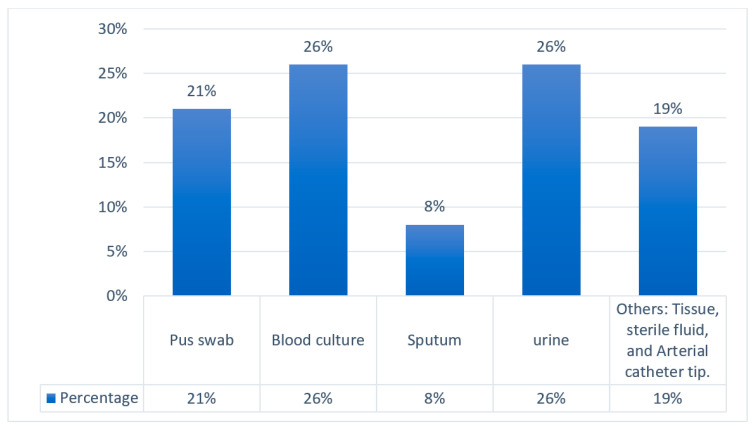
Clinical sample types and rates of CRE isolates.

**Figure 6:**
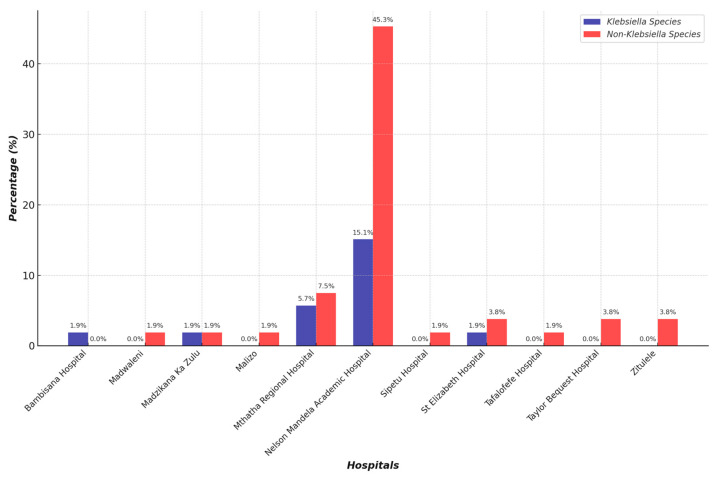
Prevalence of *Klebsiella* and non-*Klebsiella* isolates in all 11 hospitals included in this study.

**Figure 7:**
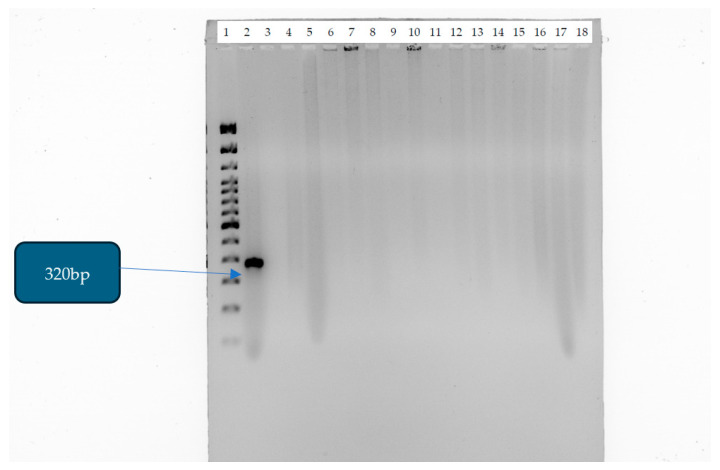
Gel electrophoresis of *mcr-1* gene (320 bp) encoding for colistin resistance. Lane 1: 100 bp DNA molecular weight ladder; lane 2: *mcr-1* positive control (amplicon size: 320 bp); lane 18: negative control: *Escherichia coli* ATCC 25922.

The distribution of CRE isolates in different age groups is represented in a graph in Figure 3. A total of 40% of CRE infections or cases were from neonates, 36% from adults, 9% from old age, and 6% from pediatrics. The pie chart in Figure 4 illustrates the relative number of CRE isolates from different wards. Each segment represents different wards, with the relative number of CRE isolates collected from each ward. Figure 5 shows the clinical sample types and rates of CRE isolates. This study included the analysis of the prevalence of *Klebsiella* and non-*Klebsiella* isolates in all 11 hospitals is shown in Figure 6; 15% of *Klebsiella* isolates or infections were reported in Nelson Mandela Academic Hospital (NMAH), followed by 5.7% in Mthatha Regional Hospital (MRG). The gel electrophoresis of *mcr-1* gene (320 bp) encoding for colistin resistance is shown in Figure 7. Table 2 shows the colistin MIC of all seven (13%) resistant CRE detected in this study.

Table 3 provides a detailed analysis of the colistin minimum inhibitory concentrations (MICs) for all seven of the 53 (13%) colistin-resistant carbapenem-resistant *Enterobacterales* (CRE) isolates de-tected in this study. This table highlights the detected MIC values of colistin resistance among the CRE isolates.

## 3. Discussion

A colistin resistance of 8 μg/mL was observed in *Klebsiella pnuemoniae* from the neonatal ICU and *Enterobacter cloacae* from the female medical ward; these isolates were obtained from the Nelson Mandela Academic Hospital (NMAH). A resistance of 16 μg/mL was found in *Serratia marcescens* isolated from a blood culture in the neonatal wards from the NMAH and Mthatha Regional Hospital (MRH), respectively. The resistance of 16 μg/mL was found also in *Serratia marcescens* from a pus swab in pediatric high care at NMAH. Lastly, *Enterobacter aerogenes* was found in urine specimens in the female ward of NMAH. *S. marcescens* is an opportunistic pathogen in hospital settings. NMAH had the highest percentage of CRE infections. The prevalence of *Klebsiella* infections (isolates) and non-*Klebsiella* infections was analyzed in all hospitals included in this study; 15.1% of *Klebsiella* infections were recorded at NMAH, with a statistical significance of 0.01. Prevalence was measured according to the level of the healthcare facility, referral status, and the level of complicated cases in which patients have already received antibiotics from lower-level care. NMAH has the highest number of infections because it is a central provincial hospital, followed by the Mthatha Regional Hospital and district hospitals.

In this study, a high number of CRE cases were recorded in neonatal wards. The high prevalence of CRE in neonate wards is concerning due to the vulnerability of this patient population. Neonates in neonatal high care units or neonatal intensive care units (NICUs) are at higher risk for CRE infections due to several key factors. They have underdeveloped immune systems, making them more susceptible to infections. They require long hospital stays in NICUs, where they are exposed to hospital-acquired pathogens. Furthermore, neonates are extremely vulnerable to CRE infections since they are frequently exposed to antibiotics and invasive procedures [17]. The prevalence of *Klebsiella* infections (isolates) and non-*Klebsiella* infections was analyzed in different age groups. The *p* values for each group were above 0.05; there was no statistical difference between the age groups.

A total of 13% colistin resistance was detected in this study. The 13% colistin resistance rate in this study is a significant concern, signaling a need for enhanced surveillance and improved antibiotic stewardship to combat resistance. The continuous tracking of colistin resistance is essential to inform treatment guidelines and public health strategies. Further studies should explore genetic mechanisms driving resistance, including the whole-genome sequencing of resistant strains. Resistance was detected in *Klebsiella pneumoniae*, *Escherichia coli*, *Serratia marcescens, Enterobacter aerogenes,* and *Enterobacter cloacae*. A high resistance of 60% in *Serratia marcescens* could be due to an intrinsic resistance to colistin. In the study by [18], in their results of the antimicrobial susceptibility test (MIC) via commercial BMD (ComASP^TM^ colistin), 14 isolates (14%) out of 100 Gram-negative isolates were found to be resistant to colistin. A study by [19] reported 31.6% colistin resistance, with 15.5% of isolates carrying *mcr* genes being identified in diarrheal pathogens among infants, children, and adults in Bangladesh. A study by [20] in India, using the similar broth microdilution method as this study, reported a 15% colistin resistance in *E. coli, K. pneumoniae*, and *Enterobacter cloacae*, while only one *Klebsiella pneumoniae* isolate harbored the *mcr-1* gene. A total of 87% colistin susceptibility was detected in this study among the species of *Klebsiella pneumoniae*, *Escherichia coli, Serratia marcescens, Enterobacter aerogenes,* and *Enterobacter cloacae*, with an 81.5% colistin susceptibility among the *E. coli* and *K. pneumoniae* isolates observed in a similar study by [21], along with a 1.7% occurrence of the *mcr-1* gene. A high phenotypic resistance of 13% in CRE in Mthatha poses the problem of spreading. We can implement antimicrobial stewardship in the hospitals in Mthatha and surrounding hospitals, as well as introduce ceftazidime/avibactam for the treatment of CRE produced by *NDM* and *KPC* gens.

The *mcr-1* gene was not detected in the clinical isolates tested in this study. Since *mcr-1* was not detected, the colistin resistance observed in 13% of isolates may be due to chromosomal mutations (e.g., modifications in the pmrA/pmrB or mgrB genes) rather than surrounding hospital-mediated transfer. The lack of *mcr-1* suggests that resistance may be less likely to spread across bacterial populations in different settings (e.g., hospitals, communities, livestock). If resistance is due to chromosomal mutations rather than plasmid-mediated factors, there might still be treatment options, such as combination therapies. Other *mcr* variants (*mcr-2* to *mcr-10*) or novel resistance mechanisms may be present and require further genetic analysis. *mcr-1* was documented for the first time in China [22]. 

The prevalence of CRE infections was high at 60% at NMAH, 13% at MRH, and 6% at St. Elizabeth Regional Hospital, followed by 4% at Madzikane kaZulu Memorial, Taylor Bequest Hospital, and Zitulele Hospital. Tafalofefe Hospital, Zitulele, Sipetu, Bambisana, Dr Malizo Mpehle, and Madwaleni hospital had a 1% prevalence. CRE infections occur according to the level of the healthcare facility and the referral status and level of complicated cases where the patient has already received antibiotics from lower levels of care. Nelson Mandela Academic Hospital has the highest prevalence of CRE cases because it is a central hospital, and this prevalence is followed by the regional and district hospitals.

In this study, the majority of the carbapenem-resistant *Enterobacterales* (CRE) were *Klebsiella pneumoniae N* = 31 (53%), followed by *Enterobacter cloacae N* = 9 (17%), *Escherichia coli* and *Serratia marcescens N* = 4 (8%), and *Serratia fonticola*, *Enterobacter aerogenes*, *Klebsiella oxytoca*, *Citrobacter koseri*, and *Citrobacter freundii N* = 1 (2%), as shown in Figure 4. A similar study in Egypt by [16] found that the carbapenem-resistant *Enterobacterales* were in the following proportions: *K. pneumoniae* (60%), *E. coli* (18%), *P. aeruginosa* (15%), and *Citrobacter* (7%).

## 4. Materials and Methods

### 4.1. Research Design

This cross-sectional study included consecutive inpatients of any age and sex with any infection by any CRE. CRE isolates were collected from National Health Laboratories (NHLS) and sent to the Microbiology Laboratory at Walter Sisulu University (WSU) between November 2022 and December 2023. In this period, fifty-three carbapenem-resistant *Enterobacterales* species from the previous CRE main study were selected. The CRE were isolated from various sources such as blood, sputum, pus swabs, urine, tissue, sterile fluid, arterial catheter tips, and tracheal aspirate. Clinical samples were analyzed using standard microbiology laboratory techniques. Only one isolate per patient was involved.

### 4.2. Study Population and Sampling Method

**Figure 8:**
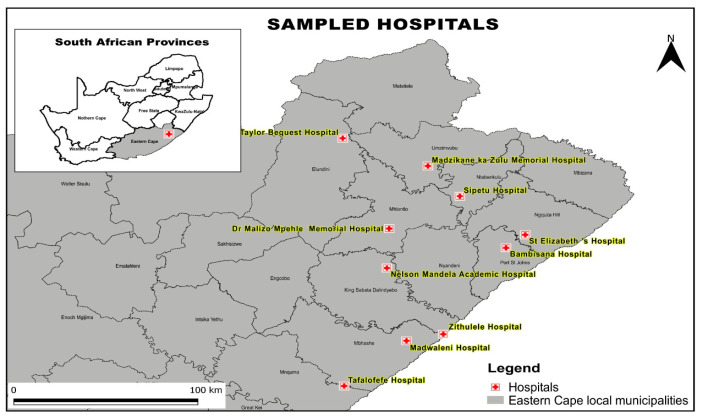
A map showing the location of hospitals covered by NHLS Mthatha [24].

The isolates were from Mthatha and surrounding hospitals. Figure 8 shows the hospitals covered by the NHLS department in Mthatha, Eastern Cape Province, as part of their routine work. The permission to use the collected CRE isolates was given to the study’s principal investigator/research supervisor by NHLS and the Mthatha Academic Hospital. The principal investigator previously collected clinical isolates used in CRE research funded by MRC in Mthatha and the surrounding hospitals. An MRC-funded project on the genes responsible for carbapenem-resistant *Enterobacterales* (CRE), epidemiology, and risk factors was conducted from 2019 to 2021 [23]. The isolates were from the following hospitals: Bambisana, Madwaleni, Dr Malizo Mpehle, Zithulele, St Elizabeth regional, Madzikane KaZulu Memorial, Taylor Bequest Hospitals, and Mthatha regional hospitals. The National Health Laboratories (NHLS) cover all the mentioned hospitals; therefore, the isolates were collected from the NHLS and sent to the Microbiology Laboratory at Walter Sisulu University (WSU) between November 2022 and December 2023. The experiment was performed at the Walter Sisulu University Microbiology Laboratory, Mthatha, Eastern Cape Province. Bacterial isolates were stored in a −80 °C freezer in skim milk, broth, and glycerol. Further tests were performed in these isolates.

### 4.3. Inclusion and Exclusion Criteria

CRE isolates were collected from the National Health Laboratory Services (NHLS) and sent to the Microbiology Laboratory at Walter Sisulu University (WSU). A CRE case was considered as carbapenem-resistant to imipenem (MIC ≥ 4), meropenem (MIC ≥ 4), Doripenem (MIC ≥ 4), or ertapenem (MIC ≥ 2) (CLSI document M100-S26) (Table 1). All carbapenem-resistant *Enterobacterales* isolates were collected and stored in skim milk media with 15% glycerol at −80 °C. CRE isolates were taken from patients (encompassing non-duplicated bacterial isolates from different specimens and duplicated isolates from different specimens with different antibiograms) that did not meet the exclusion criteria (any organisms outside the *Enterobacterales* family, any *Enterobacterales* species other than CRE, and duplicated isolates from different specimens with the same antibiogram).

Table 4 describes Carbapenems MIC breakpoints to determine their interpretation as Susceptible, Intermediate and Resistant.

### 4.4. Sampling Method and Size

The current colistin study is a sub-study of the main CRE study performed by Prof. Vasaikar from 2019 to 2021. For the present colistin study, a total of fifty-three carbapenem-resistant *Enterobacterales* species were isolated from the main study. The specimens included were taken from blood culture, sputum, pus swabs, urine, tissue, sterile fluids, arterial catheter tips, and tracheal aspirate.

### 4.5. Data Collection

The CRE isolates collected by the research supervisor of this study were transported from the NHLs in polystyrene cooler boxes to the Microbiology Laboratory at Walter Sisulu University with approval from Walter Sisulu University Health Research Ethics and Bio-safety Committee (080/2017) and the Eastern Cape Health Research Committee (EC_201710_010). For the main CRE study conducted in 2019–2021, ethical approval was granted by the Health Research Ethics and Biosafety Committee of the Walter Sisulu University (WSU), certificate number 022/11, and the Nelson Mandela Academic Hospital Ethics Committee (NMAH), Mthatha, ECP.

The research supervisor compiled the clinical records of CRE cases, including demographics, organism(s) isolated, antimicrobial susceptibility results, and medical records. Patients’ names and surnames (personal information, including the patient identity number) were removed, and new files were created with only CRE case information. CRE numbers were allocated to each strain. New files were stored on a Microsoft Excel spreadsheet in a password-protected folder on a password-protected laptop. The only individuals with access to this are the researcher and research supervisor.

### 4.6. Data Analysis

The continuous data are presented as means ± standard deviations, whilst categorical data are presented as proportions (%). The chi-square test was used to measure the difference between the two means, while the analysis of variance (ANOVA) measured the differences between groups. All the tests were 2-sided and a *p*-value < 0.05 was considered statistically significant. The analysis was performed using SPSS version 23.

### 4.7. Microbiological Investigation

#### 4.7.1. Bacterial Isolation and Identification

Identification and antimicrobial susceptibility tests were carried out in National Laboratory Health (NHLs) using BioMérieux Vitek-2 (bioMérieux, Marcy l’Etoile, France) according to the guidelines of the Clinical and Laboratory Standards Institute (CLSI) [26]. Upon arrival at the Microbiology Laboratory, the CRE isolates were streaked on McConkey agar and incubated at 37 °C for 24 h to obtain a pure culture. The isolated strains were then stored in 5 mL cryovial skim milk media with 15% glycerol at −80 °C for further investigation.

#### 4.7.2. Carbapenemase Confirmatory Tests

The detection of OXA-48, KPC, NDM, and VIM carbapenemases genes was completed using RESIST-4 O.K.N.V [27] (Coris BioConcept, Gembloux, Belgium) (immunochromatographic lateral flow assay), as per the manufacturer’s manual. This is a double immunochromatography test for the identification of the OXA-48, KPC, NDM, and VIM families of carbapenemases in bacterial culture. Each pouch contains 2 lateral flow cassettes for the identification of KPC and NMD carbapenemases and a second cassette for the identification of NDM and VIM carbapenemases. The first cassette contains monoclonal antibodies directed against KPC and OXA-48 carbapenemases; the second cassette contains monoclonal antibodies directed to NDM and VIM carbapenemases. So, when the provided buffer containing the resuspended bacteria comes into contact with the strip, the solubilized conjugates migrate with the sample. If the sample contains a KPC, OXA-48, NDM, or VIM carbapenamase, the complexes made of the conjugates will form.

#### 4.7.3. Antimicrobial Susceptibility Testing of Colistin Using ComASP Colistin 0.25–16 mg/L (LIofilchem^®^ srl, Roseto degli Abruzzi, Italy) Method

Colistin susceptibility testing was performed using BMD according to the CLSI and using ComASP™ colistin (LIofilchem^®^ srl) according to the manufacturer’s recommendations. The susceptibility test results were interpreted according to EUCAST v.12 breakpoints (2021) [28].

#### 4.7.4. Detection of Mobile Colistin Resistance (*mcr-1*) Gene

##### DNA Extraction

DNA was extracted from overnight colonies of a bacterial culture grown on fresh McConkey agar plates. To separate the cell debris and supernatant, the colonies were resuspended in Roche MagNA Pure Bacteria Lysis Buffer, vortexed, heated to 95 °C for 10 min, and then centrifuged for 3 min at 10,000 rpm. Following the manufacturer’s instructions, 400 microliters of the supernatant (the liquid containing the DNA) was used in the MagNA Pure Compact (MPC) System (Roche Applied Science, Indianapolis, IN, USA) with an MPC Nucleic Acid Isolation Kit 1 to purify the DNA [29]. A total of 200 µL of purified nucleic acid (DNA) was kept in elution tubes at −80 °C until needed.

Selection of *mcr-1* gene Primers in Table 5 was done according to the method followed by Bir et al., (2022), also we used *Escherichia coli* NCTC 13846 (colistin-resistant harboring *mcr-1* gene, colistin MIC, 4 µg/mL) was used as positive quality control strains [20].

##### DNA Library Preparation

PCR was performed in a reaction mixture containing 5 µL of purified DNA, 12 µL of DreamTaq Green PCR Master Mix (Thermofisher Scientific, Waltham, MA, USA), 6 µL of nuclease-free water, and 0.5 µL of each Forward and Reverse primer (10 µM) [20]. The following thermal cycling conditions were used.

Table 6 shows thermal conditions used for the conventional singleplex Polymerase chain reaction (PCR) used for detection of the *mcr-1* gene for colistin resistance. 

Agarose Gel Electrophoresis was used to determine the *mcr-1* gene (320 bp) encoding for colistin resistance. The DNA band was observed on a UV Transilluminator [30].

##### Ethical Considerations

The ethical clearance for this study was obtained from Walter Sisulu University, the Health Research Ethics Committee, Nelson Mandela Academic Hospital, and NHLS in the Faculty of Health Sciences, under the ethics certificate number 065/2024.

## 5. Conclusions

In this study, we found that all *N* = 53 (100%) isolates did not have the mobile colistin resistance gene (*mcr-1*); the mechanism of colistin resistance remains unknown. In total, 13% of isolates were phenotypically resistant to colistin. The absence of *mcr-1* is a positive finding in terms of limiting resistance transmission; the 13% colistin resistance observed suggests that there are alternative resistance mechanisms at play. Further investigation is needed to understand the genetic basis of resistance and its potential clinical impact. The occurrence of colistin resistance in clinical settings of Mthatha and surrounding areas can lead to difficulties in treating infections, as well as challenging therapeutic options available to treat patients infected with these organisms. Appropriate antibiotic control, the strict implementation of antimicrobial stewardship, and the education of primary care staff will play a vital role in curbing the spread of CRE.

### Limitations

This study has some limitations. For example, the research was conducted in only fifty-three isolated areas because of the limited resources available for molecular methods.

## Figures and Tables

**Table 1:** Distribution of CRE isolates from healthcare facilities in Mthatha and surrounding areas used in this study.

CRE Isolates	Percentage
*Klebsiella pneumoniae*	53%
*Enterobacter cloacae*	17%
*Escherichia coli*	8%
*Serratia marcescens*	8%
*Serratia fonticola*	2%
*Enterobacter aerogenes*	2%
*Klebsiella oxytoca*	2%
*Citrobacter koseri*	2%
*Citrobacter freundii*	2%

**Table 2:** The analysis of the occurrence of *Klebsiella* species and non-*Klebsiella* species in different age groups.

Group	*Klebsiella* Species (%)	Non *Klebsiella* Species (%)	*p*-Value
Neonates (<28 days and infants <1 year)	10/53 (19%)	11/53 (21%)	1.00
Pediatric (>13 months–≤14 years)	0/53 (0%)	3/53 (6%)	0.24
Young Adults (>14 years–≤19 years)	3/53 (6%)	2/53 (4%)	1.00
Adults (≥20 years–<65 years)	9/53 (17%)	10/53 (19%)	1.00
Elderly ≥65 years	3/53 (6%)	2/53 (4%)	1.00

**Table 3:** Colistin MIC of resistant CRE.

Hospitals	Wards	Specimen	Organisms	Colistin MIC (µg/mL)	Interpretation
1. NMAH	Female Medical Ward A	Urine	*Enterobacter Cloacae*	8	R
2. NMAH	Female Medical Ward C	Urine	*Enterobacter aerogenes*	16	R
3. NMAH	Neonatal High Care	Blood Culture	*Serratia marcescens*	16	R
4. MRH	Neonatal Ward	Blood Culture	*Klebsiella pneumoniae*	16	R
5. MRH	Neonatal Ward	Blood Culture	*Serratia marcescens*	16	R
6. NMAH	Pediatric High Care	Pus Swab	*Serratia marcescens*	16	R
7. NMAH	Neonatal Intensive Care	Pus Swab	*Klebsiella pneumoniae*	8	R

**Table 4:** Carbapenem MIC breakpoints (CLSI document M100-S26- performance standards for antimicrobial susceptibility testing, 27th edition) [25].

	Current MIC Breakpoints (µg/mL)		
	MIC Interpretation		
Carbapenems	Susceptible	Intermediate	Resistant
Doripenem	≤1	2	≥4
Ertapenem	≤0.5	1	≥2
Imipenem	≤1	2	≥4
Meropenem	≤1	2	≥4

**Table 5:** *mcr-1* forward and reverse primers.

Target Gene	Positive Control	Primer	Base Pair	Reference
*mcr-1* gene	*Escherichia coli* *13846*	F:5/-AGTCCGTTTGTTCTTGTGGC-/3 R:5/-AGATCCTTGGTCTCGGCTTG-/3	320 bp	[20]

**Table 6:** Thermal cycling conditions.

Step	Temperature (°C)	Time	Cycles
Activation	94	15 min	1
Denaturation	94	30 s	25
Annealing	58	1 min 30 s	
Elongation	72	1 min	

## Data Availability

The original contributions presented in this study are included in the article. Further inquiries can be directed to the corresponding author.
